# Body composition analysis, anthropometric indices and lipid profile markers as predictors for prediabetes

**DOI:** 10.1371/journal.pone.0200775

**Published:** 2018-08-16

**Authors:** Vineetha K. Ramdas Nayak, Kirtana Raghurama Nayak, Sudha Vidyasagar, Asha Kamath

**Affiliations:** 1 Department of Physiology, KMC Manipal, Manipal Academy of Higher Education, Karnataka, India; 2 Department of Medicine, KMC Manipal, Manipal Academy of Higher Education, Karnataka, India; 3 Department of Statistics, PSPH, Manipal Academy of Higher Education, Karnataka, India; Temple University School of Medicine, UNITED STATES

## Abstract

**Objectives:**

To compare different anthropometric indices, Body composition analysis and lipid profile markers in terms of their ability to predict prediabetes (PD).

**Methods:**

We enrolled 83 subjects with PD and 84 normoglycemic subjects who were matched for age and gender. The diagnosis of prediabetes was done according to the American Diabetes Association (ADA) criteria. All subjects were aged between 30–55 years of age and visited the outpatient department of tertiary care hospital. Anthropometric and lipid profile measurements were obtained. Analysis of body composition was done using Bodystat 1500MDD Instrument. Backward logistic regression was performed for detecting the predictors of PD. A receiver operator characteristic curve (ROC) with area under curve (AUC) was utilized for the accuracy of the predictors of PD.

**Results:**

Comparison of anthropometric measurement and body composition analysis parameters between the two groups showed that Waist circumference (WC), Body mass index, Body Fat% were significantly higher whereas Extracellular water and Dry lean weight in percentage (ECW% and DLW%) were found to be lower in PD (*p*< 0.05). Higher triglyceride (TG) levels and lower high-density cholesterol (HDL-C) with high TG/HDL-C were seen in subjects with PD. Backward logistic regression analysis found the combination of Body Fat % with WC, TG, ECW% and DLW% as strong predictors of PD. In ROC analysis, ECW% (AUC = 0.703) was the most predictive measure, followed by WC (AUC = 0.702).

**Conclusion:**

This study demonstrated that estimation of Body Fat % combined with waist circumference, Extracellular water and Dry lean weight in percentage are valuable in screening and diagnosis of prediabetes. Plasma levels of TG in lipid profile measurements can also serve as an additional marker for prediction of prediabetes.

## Introduction

According to American Diabetes Association, Type 2 Diabetes mellitus (T2DM) is a “group of metabolic diseases characterized by hyperglycemia resulting from defects in insulin secretion, insulin action or both”. The microvascular (retinopathy, nephropathy, neuropathy) and macrovascular complications (ischaemic heart disease, stroke, peripheral vascular disease) as a consequence to T2DM can result in significant morbidity and reduced quality of life [1].

India is a global hub of T2DM with an estimated population of 61.3 million patients [2]. “Prediabetes (PD), is characterized by blood glucose concentrations higher than normal, but lower than diabetes thresholds and this state is at a higher risk for development of overt T2DM” [3, 4]. Categories of PD include impaired fasting glucose (IFG) and impaired glucose tolerance (IGT), both of which can appear in isolation or in combination (combined glucose intolerance; IFG+IGT) [1]. A population based study done by Indian Medical Association in 2011 estimated that India had 62.4 million and 77.2 million people with T2DM and PD respectively [5]. Prevalence of T2DM and PD are increasing worldwide and experts have projected excess of 366 million people will have T2DM and 470 million people will have PD by 2030 [3, 6]. According to the national Indian Council of Medical Research-India DIABetes study, overall prevalence of PD in Karnataka was 10.3% [7].

The epidemiological studies on progression of IFG and IGT to T2DM indicate that over an observational period of 3–5 years, an average of 25% of subjects with IFG or IGT progress to T2DM, whereas about 50% remain in abnormal glycemic status and 25% revert to normal [8].

Indian diabetics manifest T2DM a decade earlier than their western counterparts [9]. The South Asian Indians experience greater PD with greater transition to overt diabetes compared to European countries [10]. The association of truncal obesity and T2DM based on anthropometric measurements such as waist circumference (WC) has been documented [11]. Considering Body mass index (BMI) as a measure of obesity, it has been found that T2DM occurs in Indians even with lower BMI [12]. The ectopic fat in non-adipose tissue such as skeletal muscle and liver that plays a significant role in pathogenesis of T2DM cannot be quantified with BMI as a measure [13]. The abnormal lipid metabolism with dyslipidemia that is characteristic of T2DM is also seen in PD [12, 14]. The association of WC with plasma triglyceride levels was more strongly associated with prevalence of PD and T2DM [15].

Body composition assessment based on Bioelectric impedance analysis(BIA) is a valid tool for quantification of Total body water(TBW), fat and fat-free mass or lean body mass(LBM) in the human body [16]. Estimation of body composition would be well-suited to identify the obesity predictors closely associated with the state of PD.The aim of this study was to identify indexes which would predict the risk factors for PD based on anthropometry, body composition and lipid profile markers.

## Materials and methods

### Study design and sample

This Case Control Study was conducted in the Departments of Physiology and General Medicine, at a tertiary care hospital in southern India in the time period of January 1^st^ to October 30^th^ 2016. The project protocol was approved by Kasturba Medical College and Kasturba Hospital, Manipal Institutional Ethics Committee (IEC 844/2015). The study population comprised of the adults aged 30–55 years.83 cases and 84 controls who were matched for age and gender were recruited from the outpatient clinic of General Medicine. The potential subjects were identified based on diagnostic tests and recruited by the consulting physician. A minimum of sample size with 82 participants was estimated for each group in order to detect a difference of two units in indices to be clinically significant with a power of 80% at 95% confidence interval anticipating a standard deviation of 4.6.

#### Inclusion criteria

A subject with PD was defined as someone who fulfilled the ADA criteria [17].

- Impaired fasting glucose (IFG): Fasting Plasma Glucose(FPG) between 100 and 125 mg/dl (5.6–6.9 mmol/l) OR
- Impaired glucose tolerance (IGT): 2-h post-load glucose between140 and 199 mg/dl (7.8–11.1 mmol/l) OR
- Glycated hemoglobin (HbA1c)between 5.7 and 6.4%

Controls were healthy volunteers with normal fasting blood glucose levels who were frequency matched by age and gender. Those willing to participate provided their written informed consent. This study was cleared by institutional ethics committee.

#### Exclusion criteria

Patients diagnosed with T2DM (FPG >126mg/dl),subjects on diet or exercise regimen, hypothyroidism and those on thiazides, steroids, diuretics or any other drugs which cause hyperglycemia were not included. Patients with major organ dysfunction, on pacemakers or any implantable device were excluded. Smokers, pregnant women and those with chronic illness with malignancy were excluded. Subjects who had undergone any major surgery or hospital admission in the preceding 6 months were excluded. This was assessed by self reporting questionnaire.

Participants were given an information sheet about the study and were allowed to clarify by asking questions. The personal right to withdraw from the study was guaranteed.

### Methods

#### Biochemical parameters

Blood samples for routine investigations were taken after a 12 hour overnight fasting. Plasma Glucose, Glycated hemoglobin (HbA1c), Total cholesterol (TC), HDL Cholesterol (HDL-C), Triglycerides(TG) were estimated using Cobas 6000 c501 autoanalyzer at the central laboratory of the tertiary care Hospital accredited by National Accreditation Board of Testing and Calibration Laboratories (NABL). The normal levels of lipids were considered as per National Cholesterol Education Program Adult Treatment Panel (NCEP ATP III) classification [18].

#### Anthropometric measurements

Anthropometric measurements were recorded using standard medical equipment in subjects wearing light indoor wearing clothing. Body weight and body height were rounded off to the nearest 0.5 kg and 0.5 cm respectively after the participants took off their heavy clothes, belts and shoes. Body mass index (BMI) was calculated as body mass (kg) divided by height (m)^2^. BMI was further categorized as per Asia Pacific guidelines into lean to normal (≤22.9), overweight (23–24.9) and obese groups (≥25) [19]. Waist circumference (WC) was measured using non stretchable tape in the midway section between the least palpable rib and the iliac crest with an accuracy of 0.1 cm. Hip circumference was taken around the widest portions of buttocks [20]. Asia Pacific Criteria by Misra et al has categorized waist circumference as normal and abnormal; also identified that WC cut-offs of ≥ 90 cm in men and ≥ 80 cm in women have high odds ratio for cardiovascular risk factors[21].

#### Body composition analysis

Subjects had a non- invasive assessment of body composition analysis using Body Stat 1500MDD Instrument [22] which gives the information about the Body Fluid Compartments. This employs technique of whole body bioelectrical impedance analysis configured with two removable lead wires. Measurements were taken using a hand-to-foot tetra-polar technique with participants in the supine position. This instrument provides an independent estimate of TBW by allowing constant flow of current at 400 μA at fixed frequency of 50 KHz. LBM is then calculated from TBW using hydration fraction obtained from regression equations. Fat mass is then calculated as the difference between body weight and LBM. Extracellular water (ECW) and TBW are measured using differential impedance at 50 KHz and 200 KHz respectively; following which intracellular water (ICW) is obtained by deduction [23].

The body composition parameters obtained as Body Fat (Kg) and lean (Kg) were also expressed in Body Fat in percentage (Body Fat %) and lean in percentage (Lean %). Fat free mass index in Kg/m^2^ (FFMI) and Body fat mass index in Kg/m^2^ (BFMI) were derived from the manufacturers programmed predictive equations. The other parameters included Dry Lean weight in Kg (DLW Kg) with percentage (DLW %), Total Body water as percentage of total body weight (TBW %) further classified into Extracellular water as percentage of total body weight (ECW %) and Intracellular water as percentage of total body weight (ICW %).

### Statistical analysis

The statistical data were analyzed using the SPSS 15.0(SPSS South Asia, Bangalore). Data with normally distributed parameters are presented as means and standard deviations, whereas values for lipid profile were log-transformed because of a skewed distribution and expressed as median and interquartile range (25%-75%). Independent sample t test was adopted for comparison of differences of data with normal distribution and Mann Whitney test was used for comparison of differences for data that displayed a skewed distribution. Chi Square test was selected for comparison of proportion between two groups. Independent factors associated with PD were identified during univariate analysis. Statistical significance was set at 5% level of significance.

Pearsons product moment correlation test was performed between the variables for normally distributed parameters and Spearman Rank correlation test was carried out for association of skewed variables within the groups. When the correlations were significant in both groups, Z test was adopted to compare the correlation coefficients between PD and control groups with value of significance at 0.05 level.

The main objective of the analysis was to identify the adiposity indices that would predict PD based on anthropometry, body composition and lipid profile measures. With an odds ratios(ORs) and 95% confidence intervals(95% CIs), a stepwise backward logistic regression analyses were performed to investigate the association between PD as the dependent variable and statistically significant dichotomized variables obtained from univariate analyses. The variables entered on step 1 of analyses were WC category, BMI category, Body Fat %, DLW %, ECW %, TG, HDL-C, TC/HDL-C and TG/HDL-C. The final model that stood fit as per Hosmer-Lemeshow test (H&L test) with significance was reported.

A receiver operating characteristic (ROC) curve analysis was used to assess the accuracy of predictions for PD. The accuracy was measured using the area under the ROC curve (AUC) with the 95% confidence interval (CI) expressing the sensitivity and specificity of each adiposity index as a predictor of PD. A p-value of less than or equal to 0.5 was considered statistically significant. AUC values were interpreted as acceptable discrimination for values 0.7 ≤ AUC < 0.8; as excellent discrimination for values 0.8 ≤ AUC < 0.9 and outstanding discrimination for values AUC ≥ 0.9[24].

## Results

### Comparison of anthropometric measurements, body composition parameters and lipid profile in PD and controls

Table 1 summarizes the baseline characteristics, anthropometric indices and lipid profile measurements of the subjects. Of the 167 participants, 52% were men and 48% were women, the mean age was 46.3±7.3 years. There were no statistically significant differences in age, gender, and educational level between the subjects with PD and controls. The results from these statistical interactions suggest that there are differences between the anthropometric measurements of WC, Hip C and BMI between subjects with PD and controls. The subjects with PD had a significantly higher representation in abnormal category of WC but not in WHR. Also, there was a significantly higher representation in overweight and obese categories of BMI for subjects with PD. The mean values of Body Fat %, BFMI were significantly higher while TBW % and ECW % were significantly reduced in subjects with PD. In our study, mean value of HDL-C for cases was lower than controls while TG was significantly raised in the cases. TC/HDL-C and TG/HDL-C were significantly higher in the group with PD.

**Table 1:** General, anthropometric, body composition and metabolic characteristics of the PD and control groups.

Characteristic	PD^a^ n = 83	Control n = 84	p value
Age (years)^b^	46.5±7.3	46.2±7.6	0.850
Gender(% male)^c^	51.8(43/83)	52.4(44/84)	0.941
WC(cm)^b^	96.4±9.9	89.7±10.1	**<0.001**
Hip C (cm) ^b^	101.1±8.6	93.9±9.0	**<0.001**
WHR ^b^	0.95±0.06	0.95±0.05	0.892
BMI(Kg/m^2^) ^b^	28.1±3.9	25.4±3.5	**<0.001**
WC category (% abnormal) ^c^	90.4(75/83)	61.9(52/84)	**<0.001**
WHR category((% abnormal) ^c^	97.6(81/83)	97.6(82/84)	0.990
BMI category- lean and normal (%)^c^	7.2(6/83)	22.6(19/84)	**<0.001**
BMI category- overweight (%)^c^	15.7(13/83)	25(21/84)	
BMI category- obese (%)^c^	77.1(64/83)	52.4(44/84)	
Body Fat (Kg) ^b^	25.0±8.7	20.1±6.0	**<0.001**
Body Fat % ^b^	32.9±8.2	29.8±7.6	**0.011**
Lean (Kg) ^b^	49.2±12.8	47.1±9.7	0.247
Lean % ^b^	66.8±8.3	70.2±7.6	**0.006**
DLW (Kg) ^b^	12.1±4.6	13.2±3.0	**0.085**
DLW (%)^b^	0.16±0.05	0.20±0.04	**<0.001**
TBW % ^b^	49.6±4.8	52.9±5.7	**<0.001**
ECW% ^b^	22.4±2.8	23.5±2.0	**0.004**
ICW% ^b^	27.5±3.8	29.1±4.2	**0.013**
BFMI(Kg/m^2^) ^b^	9.4±3.1	7.7±2.5	**<0.001**
FFMI(Kg/m^2^) ^b^	18.4±3.5	17.7±2.7	0.189
TC(mg/dL)^d^	195.0(170.0–221.0)	188.5(172.0–217.0)	0.820
TG(mg/dL) ^d^	148.0(106.0–189.0)	105.0(84.0–143.3)	**<0.001**
HDL-C(mg/dL) ^d^	39.0(33.0–50.0)	45.0(39.0–55.0)	**0.001**
LDL-C(mg/dL) ^d^	120.0(107.0–144.0)	119.0(107.0–145.5)	0.943
TC/HDL-C ^d^	5.0(4.2–5.8)	4.2(3.3–5.2)	**0.002**
TG/HDL-C ^d^	3.9(2.5–5.3)	2.3(1.6–3.4)	**<0.001**
FBG(mg/dL) ^d^	109.0(104.0–111.0)	92.0(89.0–95.8)	**<0.001**
HbA_1_c ^d^	5.9(5.7–6.0)	5.3(5.0–5.4)	**<0.001**

^a^ PD, Prediabetes based on HbA1c 5.7–6.4%

^b^Values expressed as mean± standard deviation p value <0.05 considered significant based on Independent sample t test.

^c^Data expressed as %(number/total) p value <0.05 significant based on Chi squared test

^d^ Values expressed as median(interquartile range) p value <0.05 considered significant based on Mann Whitney U test p values <0.05 highlighted in bold.

Abbreviations: WC, waist circumference; Hip C, hip circumference; WHR, waist to hip ratio; BMI, body mass index; WC Category, waist circumference category; Hip C Category, hip circumference category; WHR Category, waist to hip ratio category; Body Fat%, fat as% body weight; Lean%, lean as% body weight; DLW%, dry lean weight as% body weight; TBW%, total body water as % body weight; ECW%, extracellular water as% body weight; ICW%, intracellular water as% body weight; BFMI, body fat mass index; FFMI, fat free mass index; TC, total cholesterol; TG, triglycerides; HDL-C, high density cholesterol; LDL-C, low density cholesterol; TC/HDL-C, total cholesterol/high density cholesterol; TG/HDL-C, triglycerides/ high density cholesterol; FBG, Fasting blood glucose; HbA1c, Glycated hemoglobin.

### Correlation between anthropometric measurements, body composition parameters and lipid profile in PD and controls

There was a significant positive correlation for WC with BMI and Lean (Kg), whereas a negative correlation was observed for WC with ECW% and HDL-C. Lean (Kg) had a negative correlation with HDL-C. However, Lean (Kg) showed a positive correlation with TG and BMI. BMI had a negative correlation with DLW% and ECW%. TG was also found to have significant negative correlation with HDL-C. These correlations were found to be significant in PD and control groups (Tables 2 & 3).

**Table 2:** Correlation between anthropometric measurements, body composition parameters and lipid profile in PD group (n = 83).

Correlation between variables		Lean (Kg)	ECW%	BMI	TG	HDL-C	DLW%
WC	r^a^/ ρ^b^	.376**	-.281*	.663**	.011	-.268*	-.151
	p	.000^a^	.010 ^a^	.000 ^a^	.919 ^b^	.014 ^b^	.172 ^a^
Lean (Kg)	r^a^/ ρ^b^		-.247*	.271*	.265*	-.289**	.572**
	p		.024 ^a^	.013 ^a^	.016 ^b^	.008 ^b^	.000 ^a^
ECW%	r^a^/ ρ^b^			-.450**	.086	.121	-.109
	p			.000 ^a^	.439 ^b^	.275 ^b^	.325 ^a^
BMI	r^a^/ ρ^b^				.024	-.268*	-.222*
	p				.827 ^b^	.014 ^b^	.044 ^a^
TG	r^a^/ ρ^b^					-.361**	.064
	p					.001 ^b^	.567 ^b^
HDL-C	r^a^/ ρ^b^						-.090
	p						.418 ^b^

** Correlation is significant at 0.01 level (2-tailed)

* Correlation is significant at 0.05 level (2-tailed) based on Pearson ^a^ or Spearman ^b^ correlation test. Abbreviations: WC, waist circumference; ECW%, extracellular water as% body weight; BMI, body mass index; TG, triglycerides; HDL-C, high density cholesterol; DLW%, dry lean weight as% body weight, r, Pearson correlation coefficient; ρ, Spearman correlation coefficient; p, value of significance.

**Table 3:** Correlation between anthropometric measurements, body composition parameters and lipid profile in control group (n = 84).

Correlation between variables		Lean (Kg)	ECW %	BMI	TG	HDL-C	DLW%
WC	r^a^/ ρ^b^	.640**	-.547**	.697**	.350**	-.243*	-.114
	p	.000 ^a^	.000 ^a^	.000 ^a^	.001 ^b^	.026^b^	.302 ^a^
Lean (Kg)	r^a^/ ρ^b^		-.207	.464**	.364**	-.346**	.071
	p		.059 ^a^	.000 ^a^	.001 ^b^	.001 ^b^	.519 ^a^
ECW %	r^a^/ ρ^b^			-.642**	-.140	.008	.203
	p			.000 ^a^	.205 ^b^	.943 ^b^	.064 ^a^
BMI	r^a^/ ρ^b^				.219*	-.110	-.212*
	p				.045 ^b^	.318 ^b^	.050 ^a^
TG	r^a^/ ρ^b^					-.558**	-.043
	p					.000 ^b^	.697 ^b^
HDL-C	r^a^/ ρ^b^						.114
	p						.302 ^b^

** Correlation is significant at 0.01 level (2-tailed)

* Correlation is significant at 0.05 level (2-tailed) Pearson^a^ or Spearman^b^ correlation test.

Abbreviations: WC, waist circumference; ECW%, extracellular water as % body weight; BMI, Body mass index; TG, triglycerides; HDL-C; high density cholesterol; DLW%, dry lean weight as% body weight, r, Pearson correlation coefficient; ρ, Spearman correlation coefficient; p, value of significance.

In addition, the PD group alone displayed a significant negative correlation for Lean (Kg) with ECW% and a positive correlation for Lean (Kg) with DLW%. BMI also had a negative correlation with HDL-C in PD. In control group, TG had a positive correlation with WC and BMI (Tables 2 & 3).

Based on Z test, the correlation coefficients were found to be significantly stronger in control group compared to PD for WC with Lean (Kg), WC with ECW%, BMI with ECW% and TG with HDL-C. (Table 4). Though Lean (Kg) was found to have a significant correlation with HDL-C and BMI in both PD and control groups separately, there was no significant difference between correlation coefficients of the groups. Similarly, the correlation coefficients of WC with BMI and HDL-C, BMI with DLW%, TG with Lean (Kg) and HDL-C were not found to be significant between the two groups (Table 4).

**Table 4:** Comparison of correlation coefficients of anthropometric measurements, body composition parameters and lipid profile between PD and control groups.

Correlation of variables	z value	p value
WC with Lean (Kg)	-2.302	0.011*
WC with ECW%	2.064	0.02*
WC with BMI	-0.057	0.477
WC with HDL-C	-0.17	0.433
Lean(Kg) with BMI	-1.424	0.077
Lean(Kg) and HDL-C	-0.402	0.344
BMI with ECW %	2.173	0.015*
BMI with DLW%	-0.08	0.468
TG with Lean (Kg)	-0.698	0.243
TG with HDL-C	1.710	0.05*

*Differences in correlation coefficients are significant at 0.05 level (2 tailed) based on z test.

Abbreviations: PD, prediabetes; WC, waist circumference; ECW%, extracellular water as% body weight; TG, triglycerides

HDL-C: high density cholesterol; BMI, body mass index; DLW%, dry lean weight as percentage of body weight.

### Logistic regression analyses for predictors of PD based on anthropometric measurements, body composition parameters and lipid profile measures

Table 5 shows the results of final model of Backward logistic regression consisting of Body Fat %, WC category, DLW%, ECW% and TG that stood fit as per H & L test with significance. TG got preference over TG/HDL-C during selection in final steps of regression. BMI category and WHR were not found in the final model of regression analysis.

**Table 5:** Association of PD with anthropometric indices, body composition parameters and lipid profile.

Variable	B	S.E.	Wald	df	Sig.	Exp(B)	95% C.I. for Exp(B)	
							Lower	Upper
Body Fat %	0.097	0.047	4.233	1	0.040	1.102	1.005	1.209
WC category	-1.178	0.515	5.239	1	0.022	0.308	0.112	0.844
DLW %	30.993	8.428	13.523	1	0.000	2.883E13	1.932E6	4.304E20
ECW %	0.411	0.113	13.305	1	0.000	1.508	1.209	1.881
TG	-0.013	0.003	13.516	1	0.000	0.988	0.981	0.994
Constant	-15.328	4.927	9.680	1	0.002	0.000		

All the variables were subjected to backward logistic regression using Hosmer-Lemeshow test.

Abbreviations: PD, prediabetes; WC Category, waist circumference category; Body Fat%, fat as% body weight; DLW%, Dry lean weight %;ECW%, Extracellular water as% body weight; TG, triglycerides; HDL-C, high density cholesterol; B, Regression Co efficient; S.E, Standard error; df, degrees of freedom; Sig, Value of significance; Exp (B), Exponential of B or Odds Ratio; C.I,Confidence Interval.

### ROC curves for assessing accuracy of predictors of PD

**Fig 1:**
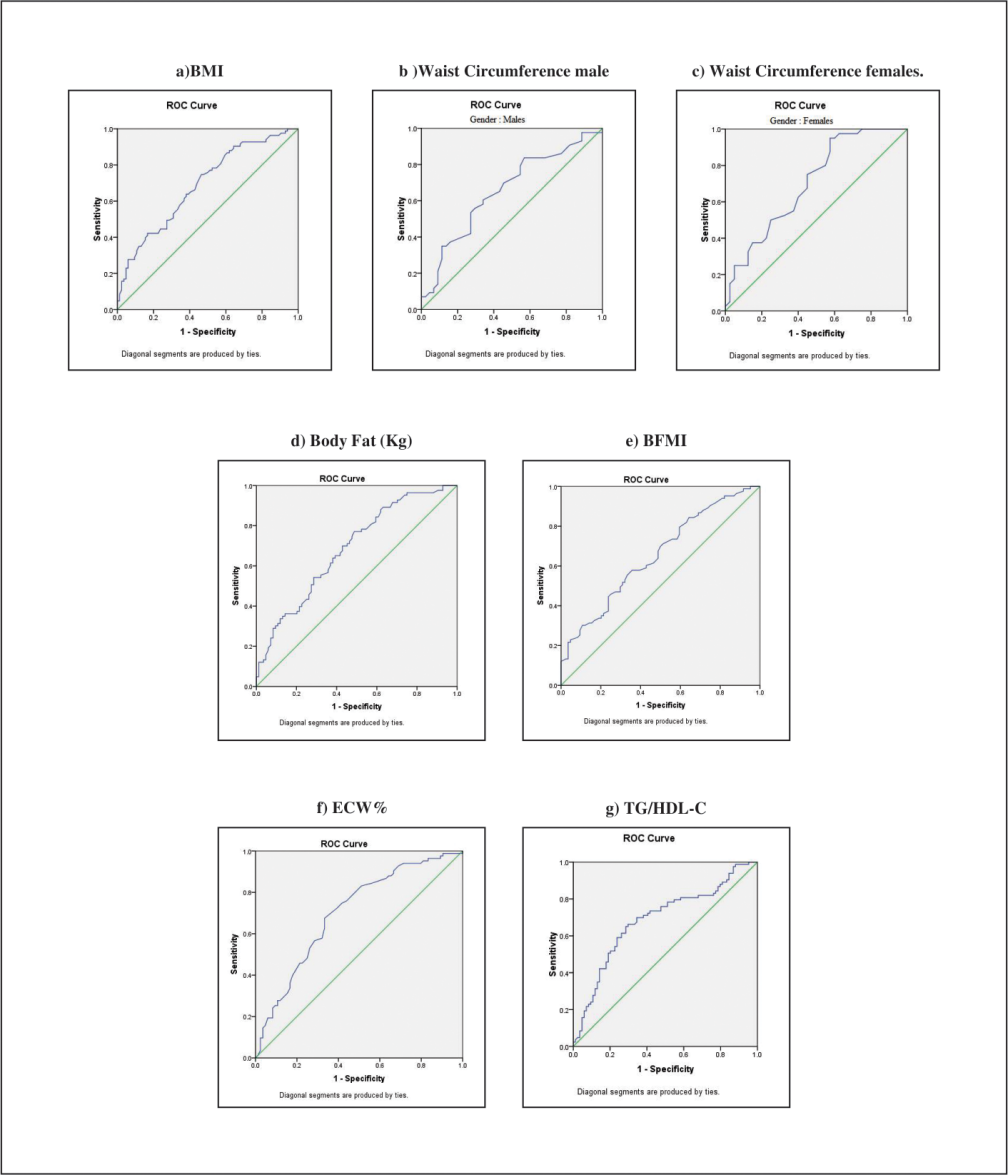
ROC curves of BMI, WC, Body Fat (Kg), BFMI, ECW% and TG/HDL as predictors of prediabetes.

Table 6 shows AUC, optimal cutoffs, and measures of diagnostic accuracy for selective anthropometric and body composition variables (AUC ≥ 0.6). ECW % and WC category in females demonstrated acceptable discrimination in ROC with value >0.7 in AUC. The BMI and TG/HDL-C had the highest sensitivity (>69%), while only ECW % showed specificity of 67%. The ROC curves for the risk factors of PD are shown in Fig 1.

**Table 6:** Diagnostic accuracy of anthropometric measurements, body composition analysis parameters and lipid profile measures for PD.

Characteristics	AUC	Optimal Cut off	Sensitivity (%)	Specificity (%)
**WC in Males**	0.656	≥94.5	61.4	61
**WC in females**	0.702	≥90.5	65	63.2
**BMI**	0.691	≥25.8199	69.9	53.6
**Body Fat(Kg)**	0.685	≥21.25	65.1	58.3
**Body Fat %**	0.603	≥29.5	62.4	59.4
**ECW %**	0.703	≤22.550	62.7	67
**BFMI**	0.652	≤7.650	63.9	51.2
**TG/HDL-C**	0.687	≥2.95	69.9	65.5

The optimal cutoff points were chosen based on the maximization of the sensitivity and specificity product.

Abbreviations: PD, prediabetes; WC, waist circumference; Body Fat%, fat as% body weight; BMI, body mass index; ECW%, extracellular water as% body weight; BFMI, body fat mass index; TG, triglycerides; HDL-C, high density cholesterol; AUC, Area under the curve.

## Discussion

In this case control study, we evaluated 83 subjects with prediabetes and 84 normoglycemic subjects to identify the anthropometric indicators, body composition variables and lipid profile markers for prediction of prediabetes. Current study demonstrated that WC, Hip C and BMI were significantly higher in subjects with PD (Table 1). Several previous studies have concluded that high WC and BMI are risk factors for PD and T2DM predisposing to cardiovascular disease with coronary artery disease [25, 26]. The abdominal obesity that is measured using WC has shown to be associated with an increased risk for coronary artery disease and T2DM, independent of overall adiposity [27]. Our results showed that 90% of subjects with PD had abnormal WC and BMI based on cut off values as per the Asia Pacific Criteria. BMI represents the total body mass while WC and WHR reflect abdominal obesity. WC, WHR are considered to be surrogate measures of visceral adipose tissue. The International Diabetic Federation considered WC as a major criterion for definition of metabolic syndrome [28]. In a recent meta-analysis, WC was said to have strong correlation with the development of PD and overt T2DM than BMI [29]. In a cross-sectional study after adjusting for potential confounding factors, participants with a visceral fat mass measured by dual-energy X-ray absorptiometry that was in the upper 10th percentile had a higher odds ratio for development of PD and T2DM [30]. Visceral adipose tissue has been shown to lead to hepatic insulin resistance through its high degree of lipolytic activity and high release of free fatty acids into portal circulation [31].The proposed reason for insulin resistance has been reported earlier as the release of adipokines such as TNF-α, IL-6 and resistin from the visceral adipose tissue [32].

Our study results showed higher body fat % with lower lean %, TBW %and ECW % in subjects with PD (Table 1). Further, the higher WC that was associated with significant increase in Lean (Kg) in normoglycemic subjects was not seen in the state of PD (Table 4).A combined state of higher body fat and lower lean mass contributes to insulin resistance and hyperglycemia by disrupting glucose uptake by muscle tissue through release of endocrine mediators by adipose tissue [33]. TBW reflects the lean soft tissue like muscle mass [34] and since lean % is reduced in PD, this could the underlying rationale for the finding of low TBW% in this study.

We obtained statistically significant higher values for TG and lower values for HDL-C in subjects with PD (Table 1). The normal glycemic state exhibited a state of high HDL-C with low TG levels whereas induction into the state of PD resulted in high TG with low HDL-C levels (Tables 2, 3 & 4). This is consistent with earlier studies which have shown that plasma levels of TC, LDL-C, TG were higher and HDL-C was lower in subjects with PD [35, 36]. The state of hypertriglyceridemia with low HDL-C in PD is of considerable concern from clinical point of view as this combination can further lead to dysregulation in glucose metabolism by inducing insulin resistance and beta cell dysfunction. The dyslipidemic state of high plasma TG and FFA levels with low HDL- C can be the consequence as well as source of hyperglycemia [37,38].A recent study has further established the onset of aberrant lipid metabolism in PD similar to that occurring in T2DM [39].

While evaluating the body composition parameters and its utility to predict PD using regression model we found that the combination of Body Fat%, WC category, DLW %, ECW%, and TG were superior in prediction of PD (Table 5). BMI or BMI category were not included in the final step of the regression analysis. Our results were consistent with an earlier study which showed that WC and not BMI to be the main predictor of insulin resistance in T2DM as per the regression model [30, 40]. This is in contrast to a previous study which stated that WC, while measuring T2DM risk depends on BMI and both should be included in risk assessment [41]. In addition to the findings from Gholi et al that WC and plasma TG levels were higher in subjects with PD [42], the significant association of TG with WC was also found to be effective to predict PD [15]. Though TG got preference over TG/HDL-C in the present study, a recent research evidence reported WC to be included in the final regression analysis with TG/HDL-C in newly diagnosed T2DM [43]. Our observation is conforming to a previous study that suggested Body Fat % to be included while measuring the risk for PD and T2DM, even in subjects with BMI less than 25 Kg/m^2^[44]. ECW % and DLW % can serve as adjunct diagnostic parameters to enhance the predictability of PD. This is a novel finding based on observation from the present study.

We analyzed the usefulness of anthropometric indices as predictors of PD using AUC of ROC curves (Fig 1). In the present study, the AUC for WC was greater than that for BMI suggests that anthropometric indices such as WC that reflect central obesity are better for predicting PD (Table 6). This is more evident in Chinese immigrants from Asian population where diabetes and IFG were reported in subjects with a normal BMI, especially among those with larger waist circumferences [45].

According to WHO Expert consultation the cut off in BMI for moderate risk for development of T2DM and cardiovascular disease ranges from 22kg/m^2^ to 25kg/m^2^ [46].The cut off values as obtained from ROC for BMI, WC for males and females were 25.81 kg/m^2^, 94.5 cm and 90.5cm respectively. A case study in Indian population revealed receiver operator characteristic curve analysis of WC cut-offs for males was 90 cm with a sensitivity and specificity of 71% and 96%, respectively, and for females was 85 cm with a sensitivity and specificity of 86% and 93%, respectively, having a positive association with metabolic syndrome [47].

From a clinical and public health perspective, it is observed that waist circumference remains an effective index of obesity. The results of our research suggest that is it reasonable that WC can be monitored to address the potential development of T2DM.Waist circumference is not only easy to obtain but also accurate marker of PD. TG among lipid profile indicators reveal significant association with PD. Body Fat %, ECW% and DLW% measured using biometric impedance analysis could be used in routine clinical practice since advanced imaging tools like Body densitometry assessment and CT scan require intensive training to be used by the healthcare provider besides adding to the cost burden of the patient [48].

## Conclusions

This study demonstrated that estimation of Body Fat %, combined with waist circumference Extracellular water and Dry lean weight in percentage are valuable in screening and diagnosis of prediabetes. TG in lipid profile measurements can serve as an additional marker for prediction of prediabetes. Further studies have to be taken up to elucidate the altered body composition in prediabetes that lead to change in body fluid compartments.

### Strengths

The study was conducted by trained interviewers and anthropometric data were obtained by repeated measurement using a standard protocol. The results of our study can be used to formulate the guidelines for anthropometric cut offs especially in the South Indian population. In a resource scarce lower middle-income country like India, incorporating the physiological aspects like Body Fat %, Extracellular water percentage and Dry Lean weight percentage along with anthropometric measurements could benefit in screening of prediabetes. This could potentially be easily and effectively used by the peripheral health workers.

### Limitation

Our observation of abnormal body composition to predict prediabetes can be used in the population screening of high risk subjects and needs to be validated in a large sample.

## Supporting information

S1 Dataset Data pertaining to subjects with prediabetes and control population. (XLSX)

S1 Appendix Proforma for case collection and screening. (DOCX)
