# Supplementary material for: Body composition analysis, anthropometric indices and lipid profile markers as predictors for prediabetes
Source: PLoS One. 2018 Aug 16;13(8):e0200775. doi: 10.1371/journal.pone.0200775 (PMC6095495; doi:10.1371/journal.pone.0200775)
Supplement: S1 Appendix — (DOCX) [file pone.0200775.s002.docx]

*PROFORMA FOR CASE COLLECTION AND SCREENING*

**A case control study of body composition , anthropometric and metabolic risk factors among pre-diabetes in a tertiary referral centre in South India**

**OP/IP Number:**

Age: _____ Sex (M/F)

4. Domicile (1=Rural / 2= Urban)

**PERSONAL AND LIFESTYLE RELATED DATA**

**P1 Education:**

1. Illiterate 2. Just literate

3. Primary school (up to 4th standard) 4. Secondary school (5th to 8th standard)

5. High school (9th and 10th standard) 6. College level but not graduate

7. Graduate or Professional 8. Postgraduate and above

9. Unknown 10. Other [Specify] ____________________

**P 2 Occupation**: Are you currently…

1. Retired 2. Unable to work because of disability 3. A student

4.Not currently working for pay (includes looking for work)

5. Working full-time (more than 35 hours a week) 6. Other ___________________

P 3 Current Marital status: 1 Single 2 Married

P 4 Years of schooling: Years

P5 Family history:

P 6 Minutes of regular physical activity:

P8 Past history of hospital admission

P 8 Alcohol consumption:  Regular  Occasional Non-drinker

P 9 Smoking:  Current  Past Never

P910 Drug Abuse  Yes  No; If Yes specify___________________

**MEASUREMENTS**

**P11** Height cm **P 12** Weight kg

P13: Body mass index

P14. Waist Hip ratio:

P15. Waist circumference (cm)

P16: Hip circumference (cm)

**RELEVANT MEDICAL HISTORY**

P 17 Concomitant disease

Diabetes mellitus Yes  No Duration in years Treatment

Hypertension  Yes  No

Hypothyroidism  Yes  No

Psychiatric illness  Yes  No

COPD  Yes  No

**INVESTIGATIONS**:

P18 Blood Glucose Fasting mg/dl

P19 Glycated Hb

P20 Lipid Profile

1. S cholesterol
2. TG
3. VLDL
4. LDL
5. HDL

P 21 Body Composition Analysis

1. Total Body Water (TBW)
2. Basal Metabolic Rate (BMR)
3. Body Mass Index (BMI)
4. Fat Free Mass (FFM)
5. Fat Free Mass Index (FFMI)
6. Body Fat Mass Index (BFMI)
7. Lean mass
8. Fat mass
9. Extracellular water
10. Intracellular water

Thank you for your participation

All information obtained in the study will be kept Confidential and used for medical research only
